# Effectiveness of an Intervention Providing Digitally Generated Personalized Feedback and Education on Adherence to Continuous Positive Airway Pressure: Randomized Controlled Trial

**DOI:** 10.2196/40193

**Published:** 2023-05-22

**Authors:** Joyca Lacroix, Jan Tatousek, Niek Den Teuling, Thomas Visser, Charles Wells, Paul Wylie, Russell Rosenberg, Richard Bogan

**Affiliations:** 1 Department of Digital Engagement, Cognition & Behavior, Philips Research, Eindhoven, Netherlands Eindhoven Netherlands; 2 Department of Remote Patient Management and Chronic Care, Philips Research Eindhoven Netherlands; 3 Philips Experience Design Eindhoven Netherlands; 4 SleepMed, Inc Macon, GA United States; 5 Arkansas Center for Sleep Medicine Little Rock, AR United States; 6 Neurotrials Research, Inc Atlanta, GA United States; 7 SleepMed, Inc Columbia, SC United States; 8 University of South Carolina School of Medicine Columbia, SC United States

**Keywords:** therapy adherence, personalized feedback, personalized education, tailored communication, psychological profile, continuous positive airway pressure therapy, CPAP therapy, obstructive sleep apnea

## Abstract

**Background:**

Many people worldwide experience obstructive sleep apnea, which is associated with medical and psychological problems. Continuous positive airway pressure (CPAP) is an efficacious therapy for obstructive sleep apnea, but its effect is limited by nonadherence. Studies show that personalized education and feedback can increase CPAP adherence. Moreover, tailoring the style of information to the psychological profile of a patient has been shown to enhance the impact of interventions.

**Objective:**

This study aimed to assess the effect of an intervention providing digitally generated personalized education and feedback on CPAP adherence and the additional effect of tailoring the style of the education and feedback to an individual’s psychological profile.

**Methods:**

This study was a 90-day, multicenter, parallel, single-blinded, and randomized controlled trial with 3 conditions: personalized content in a tailored style (PT) in addition to usual care (UC), personalized content in a nontailored style (PN) in addition to UC, and UC. To test the effect of personalized education and feedback, the PN + PT group was compared with the UC group. To test the additional effect of tailoring the style to psychological profiles, the PN and PT groups were compared. Overall, 169 participants were recruited from 6 US sleep clinics. The primary outcome measures were adherence based on minutes of use per night and on nights of use per week.

**Results:**

We found a significant positive effect of personalized education and feedback on both primary adherence outcome measures. The difference in the estimated average adherence based on minutes of use per night between the PT + PN and UC groups on day 90 was 81.3 minutes in favor of the PT + PN group (95% CI −134.00 to −29.10; *P*=.002). The difference in the average adherence based on nights of use per week between the PT + PN and UC groups at week 12 was 0.9 nights per week in favor of the PT + PN group (difference in odds ratio 0.39, 95% CI 0.21-0.72; *P*=.003). We did not find an additional effect of tailoring the style of the intervention to psychological profiles on the primary outcomes. The difference in nightly use between the PT and PN groups on day 90 (95% CI −28.20 to 96.50; *P*=.28) and the difference in nights of use per week between the PT and PN groups at week 12 (difference in odds ratio 0.85, 95% CI 0.51-1.43; *P*=.054) were both nonsignificant.

**Conclusions:**

The results show that personalized education and feedback can increase CPAP adherence substantially. Tailoring the style of the intervention to the psychological profiles of patients did not further increase adherence. Future research should investigate how the impact of interventions can be enhanced by catering to differences in psychological profiles.

**Trial Registration:**

ClinicalTrials.gov NCT02195531; https://clinicaltrials.gov/ct2/show/NCT02195531

## Introduction

### Background

Many people worldwide meet the minimal diagnostic criteria for obstructive sleep apnea (OSA). OSA is characterized by repeated obstructions of the upper airway during sleep, resulting in sleep fragmentation leading to daytime sleepiness and oxygen desaturation. Both sleep fragmentation and oxygen desaturation often go unnoticed by patients but are related to many medical, psychological, and cognitive problems [1-5]. Continuous positive airway pressure (CPAP) is an efficacious therapy for moderate to severe OSA and has been shown to positively influence daily functioning, mood, and insulin resistance and possibly reduce the risk for several chronic conditions such as cardiovascular diseases [6]. However, its effectiveness is limited by poor uptake and 30% to 60% nonadherence in treated patients [7-9]. Owing to the substantial effect of adherence levels on the quality of life, health outcomes, and health care costs of patients with OSA [10], researchers and health care centers have focused on developing effective CPAP adherence interventions. In addition, payers are stimulating adherence by making reimbursement for CPAP therapy devices dependent on patients meeting adherence criteria, for example, the Centers for Medicare and Medicaid Services (CMS) in the United States [11]. There exists a clear need for an intervention for promoting CPAP therapy that can be delivered with minimal burden in UC settings.

Qualitative studies suggest that patients with OSA are more likely to engage with CPAP if they have an understanding of how OSA may impact their health and quality of life and how CPAP works to mitigate health risks [12-14]. The literature focused on the impact of providing educational materials to stimulate CPAP adherence is inconclusive [15-20]. Although the overall effect of education alone on CPAP adherence is modest [21], research findings suggest a larger effect when education is combined with personalized feedback based on each patient’s diagnostic and therapy data [7,18,22,23].

In addition to providing education and feedback content personalized based on personal data (hereafter referred to as personalized education and feedback), health-related information can be delivered in a tailored style based on an assessment of the psychological parameters that are likely to influence how the receiver processes the information. Adopting a tailored style approach to delivering education and patient feedback has been shown to promote behavior change in a variety of therapeutic areas [24-26]; however, we are not aware of any studies in which a CPAP adherence intervention has been developed and delivered according to psychological profiles. In preparation for this study, we adopted a patient profiling approach to developing a psychological patient stratification model; a questionnaire for profiling patients as “fighters,” “analysts,” “sensitives,” or “optimists” corresponding to their motivational drivers and needs when it comes to coping with a chronic condition and therapy; and a method for tailoring health-related communication to address the drivers and needs (eg, the need for understanding all the details of the condition and being motivated by the stories of other patients who are in a similar situation) of each of the profiles both in terms of wording and visual layout. The profiling model, the questionnaire for assessing the profiles, and the method for tailoring the style of health-related education to psychological profiles were developed and validated in an iterative fashion based on qualitative and quantitative data obtained from >500 patients and >20 care professionals (Tatousek, J, Lacroix, J, unpublished data, 2010-2012).

### Goal of This Study

Our objective was to complete a 3-arm, parallel, randomized controlled trial to test the impact of personalized education and feedback, either tailored or nontailored to each individual’s psychological profile, on CPAP adherence compared with usual care (UC). We hypothesized that personalized CPAP education and feedback would be associated with increased adherence compared with UC. Furthermore, we hypothesized that personalized education and feedback delivered in a tailored style would lead to increased adherence compared with those delivered in a nontailored style.

## Methods

### Ethics Approval

Approval was sought from the Allendale Institutional Review Board (PGI-BIG4-1331-MS). This study was registered at ClinicalTrials.gov (NCT02195531). Data collection took place at 6 US sites: SleepMed, South Carolina; Sleep Med, Georgia; Pulmonary Associates, Arizona; Arkansas Center for Sleep Medicine, Arkansas; NeuroTrials Research, Georgia; and Center for Sleep Medicine, Pennsylvania. Recruitment and randomization were conducted from 2014 until 2015. All the participants provided written informed consent.

### Design

This study was a 90-day multicenter, parallel-arm, single-blinded, and randomized controlled trial of 3 conditions: personalized content in a tailored style (PT) in addition to UC, personalized content in a nontailored style (PN) in addition to UC, and UC.

### Recruitment, Baseline Assessment, Randomization, and Blinding

Participants were recruited from a sleep clinic at each site. The inclusion criteria were as follows: 21 to 80 years of age, apnea hypopnea index ≥10 events per hour, and CPAP naive. Potential participants were excluded if they had a chronic condition, including unstable depression or anxiety, dementia, a facial musculoskeletal disorder, obesity hypoventilation syndrome, central or complex sleep apnea, or had a requirement for supplemental oxygen. Before randomization, all participants were asked to complete a psychological profiling questionnaire to categorize them as a “fighter,” an “optimist,” a “sensitive,” or an “analyst” and to assess their opposite profile (ie, the profile that applied to them the least; Multimedia Appendix 1); a brief sociodemographic questionnaire; and the Epworth Sleepiness Scale (ESS) [27]. In addition, constructs from psychological theories of health behavior change (ie, the social cognitive theory [28] and the transtheoretical model [29]) were selected as secondary outcomes and assessed at baseline because they are increasingly recognized as consistent predictors of adherence to CPAP [30-33]: self-efficacy, perceived importance, outcome expectations, and motivation (using questionnaires from the study by Aloia et al [33]).

Willing and eligible participants were randomized in a 1:1:1 ratio using an adaptive biased coin randomization sequence, stratified by gender and psychological profile [34]. The randomization sequence was electronically housed and not accessible to the study staff.

Participants were blinded by not being informed of their group allocation. It was not possible to blind the study staff given the need to provide the study materials (ie, brochures) to each participant.

### Provision of UC

OSA diagnosis; the initiation of CPAP therapy, including mask fitting (day 0); and all follow-up care occurred at each sleep clinic following the usual procedures. A System One automatically adjusting CPAP device (Philips Respironics, Koninklijke Philips NV) was supplied to all the participants along with a modem for the remote monitoring of adherence, pressure delivery, and mask leak, which were accessed via the Philips Encore database. (At the time of the study, the Philips Respironics System One automatically adjusting CPAP device was a leading model in the Philips CPAP portfolio, and it was used by all the study participants. Since the time of the study, the model has been superseded by new models. At the time of the study, Philips Sleep in Respiratory Care [Respironics] was located at 1740 Golden Mile Hwy, Monroeville, PA 15146. In 2021, the company relocated to 6400 Penn Avenue, Pittsburgh, PA 15206). The device settings were determined by the referring physician.

### Adherence Intervention

The intervention comprised the delivery of personalized education and feedback aimed at increasing CPAP adherence in 2 ways.

First, the participants received 2 educational brochures: one explaining OSA delivered in the sleep clinic on day 0 (and again by mail on day 2) and another explaining CPAP therapy delivered on day 7 by mail. The brochures provided general information and aimed to stimulate perceived importance, outcome expectations, self-efficacy, and motivation to adhere to CPAP therapy by covering 4 topics, namely potential risks of untreated OSA (perceived importance and motivation), potential benefits of CPAP (perceived importance and outcome expectations), tips for getting started on CPAP easily and confidently (self-efficacy), and common pitfalls with corresponding troubleshooting recommendations (self-efficacy and motivation). Four different versions of each of the 2 educational brochures were produced, each tailored to one of the 4 psychological profiles. The contents covered in the brochures were very similar; the only difference was the tailoring to the psychological profile. Textual or visual references to age, gender, and ethnicity within the brochures were adjusted for each individual.

The second and biggest element of the intervention relied on digital technologies to compose 7 personalized CPAP reports delivered weekly by mail beginning on day 11 (refer to Multimedia Appendix 2 for examples). Weekly CPAP therapy data (on use and leakage) were collected from the participant’s CPAP device and digitally transmitted as input to an algorithm that generated the content of the weekly CPAP reports. The weekly CPAP reports contained personal feedback in the form of a summary of the patient’s adherence and leak data from the previous week as well as a recommendation for a troubleshooting action targeting a personally relevant topic. The feedback and recommendations focused on stimulating and facilitating CPAP adherence and aimed at building and maintaining perceived importance, outcome expectations, self-efficacy, and motivation by emphasizing current successes and relating to past successes (self-efficacy), repeating benefits and creating awareness of noticeable benefits (perceived outcomes and perceived importance), and offering personally relevant actionable insights and recommendations for further improvement (self-efficacy and motivation). A total of 25 recommendations for action were developed in advance, covering 4 topics: use hours, nights of use, mask fitting, and general tips. The 25 recommendations were styled in 4 ways according to the psychological profiles, resulting in a pool of 100 recommendations. The recommendations were reviewed and approved by 5 sleep physicians, who were also the principal investigators of the study. Each week, the recommendation for each patient was selected by the algorithm based on the psychological profile, adherence and leak parameters collected over the prior week, and the history of recommendations sent to that patient in previous weeks to ensure relevance and variety.

The contents of the brochures and CPAP reports were compiled into templates, each targeting one of the psychological profiles. As such, all intervention materials were digitally generated in a systematic, automated fashion. To increase the likelihood of measuring an effect of style tailoring, we maximized the tailoring difference between the PT and PN groups. The participants randomized to PT received the brochures and reports consistent with their profile, whereas those randomized to PN received materials consistent with their opposite profile. The opposite profile for each participant was determined based on the psychological profiling questionnaire.

### Assessment of Outcomes

Our primary CPAP adherence outcome variables were generated over 90 days for each group: mean hours used per night (continuous) and mean number of nights used per week (continuous). Our secondary CPAP adherence outcome was the percentage of participants reaching the CMS adherence threshold. Days of nonuse were imputed with an adherence of 0 hours per night. Some participants completed their final study visit in the final week before day 90 because of planning challenges. These participants were censored, and it was assumed that they continued with therapy as before in the final days until day 90 (ie, missing data did not contribute to the model estimates).

Subjective secondary outcomes were assessed at different time points. The self-efficacy, perceived importance, outcome expectations, and motivation questionnaires were repeated on days 11, 56, and 90, whereas the ESS was repeated on days 56 and 90. A single item assessing satisfaction with CPAP therapy on a scale of 0 to 10 was completed on days 11, 56, and 90.

### Statistical Analysis

#### Overview

Statistical analyses were conducted using R (version 3.6.3; R Foundation for Statistical Computing) [35]. To investigate the overall impact of the personalized CPAP intervention (providing personally relevant CPAP feedback and education), the PT and PN groups were combined and compared with the UC group (PT + PN vs UC). To examine the impact of tailoring the style of the personalized CPAP intervention to psychological profiles, the PT and PN groups were compared (PT vs PN). Our primary outcome variables were hours of use per night and nights of use per week. Secondary outcome variables were the percentage of participants reaching the CMS adherence threshold (ie, use of at least 4 hours per night during 70% of the days in a 30-day time window anywhere during the 90-day study duration) and the subjective outcome variables (ESS scores, outcome expectations, perceived importance, motivation, self-efficacy, and therapy satisfaction). Hours of use per night was modeled using linear mixed-effects modeling. Nights of use per week was modeled using beta linear mixed-effects modeling. The models were estimated using the *lme4* package (version 1.1-21) [36] and the *GLMMadaptive* package (version 0.6-8) [37]. Group, time, and the interaction thereof were included as fixed effects. Time was treated as a continuous variable. Each participant was modeled with random effects in the intercept and slope. Type-III Wald chi***-***square tests were used to compute the *P* values for the effects in the mixed models. All tests were considered statistically significant if *P*≤.05. In the case of a significant group-by-time interaction effect, models were rerun without interaction terms to assess the main effects. Subjective outcome variables (ESS scores, outcome expectations, perceived importance, motivation, self-efficacy, and therapy satisfaction) were modeled similarly using mixed-effects models; however, instead of time, the moment of assessment (1,2,3,4) was used. Log-rank tests were performed to determine the percentage of participants who reached the CMS adherence threshold *over* time. All analyses were performed in accordance with the intention-to-treat principle.

#### Power

In the study by Hoy et al [38], adherences of 5.4 (SD 2.7) and 3.9 (SD 3.6) hours per night were reported with an intervention of augmented support and education, respectively. On the basis of this effect size, we calculated a requirement of 114 participants to detect this effect as statistically significant (α=.05; β=.8). To cover dropouts and withdrawals, we selected a sample size of 150 participants.

## Results

### Overview

In total, 169 participants were randomized. Of these 169 participants, 3 (1.8%) participants voluntarily withdrew from the trial and requested that their data not be analyzed; therefore, the analytic sample was 166 participants (PT, n=48, 28.9%; PN, n=55, 33.1%; and UC, n=63, 38%). The descriptive characteristics of the participants are shown in Table 1. No statistically significant differences were observed between the 3 groups (all *P*>.05). Some participants had their final visit 1 or a few days before day 90 owing to the availability of participants for the final visit. This was the case for 23% (11/48), 35% (19/55), and 27% (17/63) of participants in the PT, PN, and UC groups, respectively, resulting in a total of 32 (out of 4320), 38 (out of 4950), and 46 (out of 5670) nights of missing data in the PT, PN, and UC groups, respectively.

**Table 1:** Descriptive characteristics of the participants (n=166).

			PT^a^ group (n=48)	PN^b^ group (n=55)		UC^c^ group (n=63)	*P* values	
Age (years), mean (SD)			50.5 (10.9)	53.3 (12.8)		48.0 (12.5)	.07	
Gender (men), n (%)			37 (77)	38 (69)		40 (63)	.68	
BMI (kg/m^2^), mean (SD)			32.8 (5.6)	33.6 (6.5)		34.5 (8.2)	.43	
AHI^d^ (events/hour), mean (SD)			27.1 (16.1)	28.2 (16.2)		32.7 (17.9)	.17	
ESS^e^ (on a scale of 24), mean (SD)			10.4 (5.3)	11.2 (5.6)		11.1 (5.6)	.73	
**Mask type, n (%)**								.13
	Oronasal	9 (19)		15 (27)	26 (41)			
	Nasal	23 (48)		22 (40)	22 (35)			
	Pillow	16 (33)		18 (33)	15 (24)			

^a^PT: personalized content in a tailored style.

^b^PN: personalized content in a nontailored style.

^c^UC: usual care.

^d^AHI: apnea hypopnea index.

^e^ESS: Epworth Sleepiness Scale.

### Effect of the Personalized Intervention

#### Hours of Use Per Night

**Figure 1:**
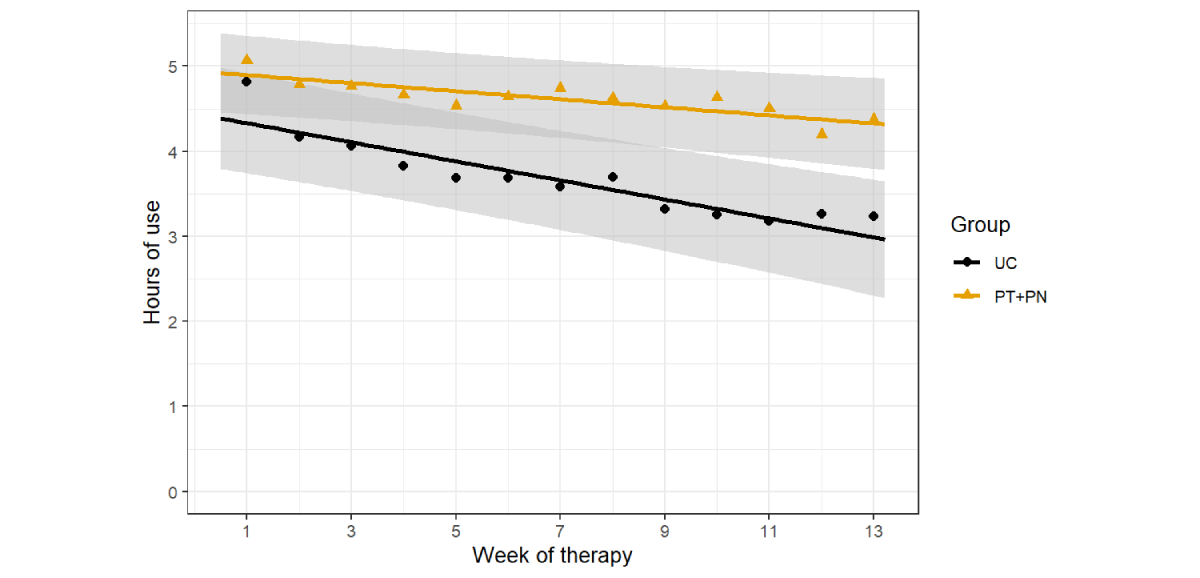
Average continuous positive airway pressure adherence (hours of use per night) in the personalized content in a tailored style + personalized content in a nontailored style (PT + PN) group and usual care (UC) group. Each data point represents the mean adherence for each 7-day period. Each line is a fitted linear model with the shaded area corresponding to the 95% CI.

Average adherence throughout the 90-day study period was 4.62 (3.15) hours per night for the PT + PN group and 3.68 (3.22) hours per night for the UC group. Figure 1 shows the average CPAP adherence in terms of hours of use per night in the PT + PN and UC groups and the fitted linear mixed model. A visual assessment of the residuals via a normal quantile plot showed that the model errors were approximately normal. The model showed a significant group-by-time interaction such that the rate of decline in adherence was significantly slower for the PT + PN group than for the UC group, with an estimated difference in slope of 0.55 minutes per night in favor of the PT + PN group (95% CI 0.09-1.02; *P*=.02). The same model, excluding the interaction term, showed a main effect of time (slope −0.62 minutes per night, 95% CI −0.85 to −0.39; *P*<.001) and a main effect of group (46.72, 95% CI 2.18-91.25; *P*=.04). The difference in the estimated average adherence based on minutes of use per night between the groups at day 90 was 81.3 minutes in favor of the PT + PN group (95% CI −134.00 to −29.10; *P*=.002).

#### Nights of Use Per Week

**Figure 2:**
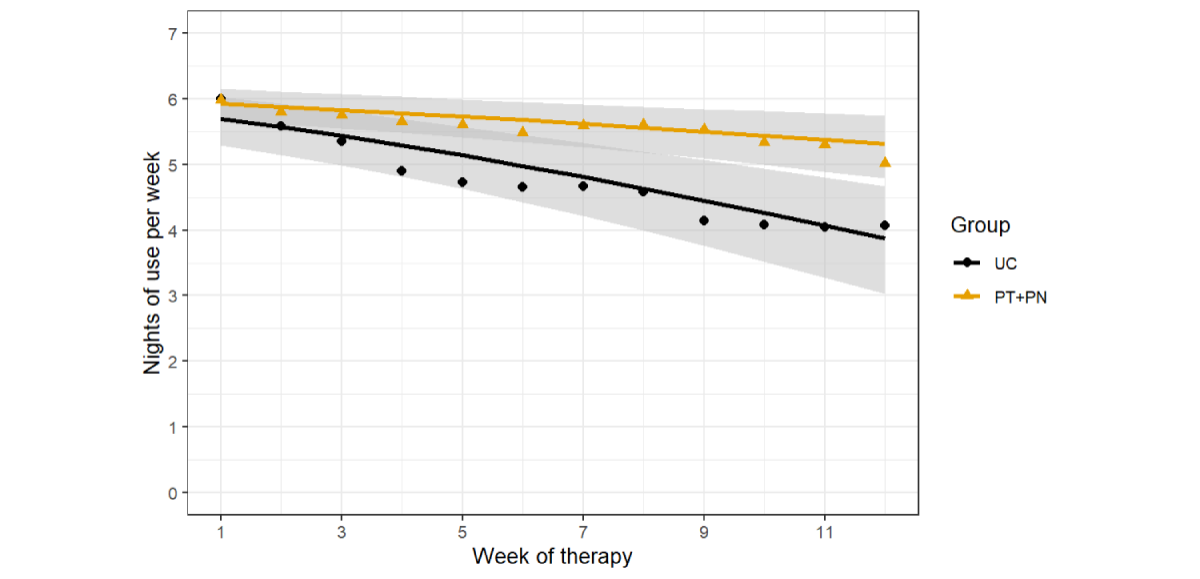
Average continuous positive airway pressure adherence (nights of use per week) in the personalized content in a tailored style + personalized content in a nontailored style (PT + PN) group and usual care (UC) group. Each data point represents the mean adherence for each 7-day period. Each line is a fitted linear model with the shaded area corresponding to the 95% CI.

The average number of nights of use per week throughout the 90-day study period was 5.6 (SD 2.2) nights per week in the PT + PN group and 4.7 (SD 2.7) nights per week in the UC group. Figure 2 shows the average number of nights of CPAP use per week for the PT + PN and UC groups. The PT + PN and UC groups had an average weekly decline of 0.05 nights per week and 0.16 nights per week, respectively. The mixed-effects beta regression model showed a significant group-by-time interaction, with a significant difference in odds ratio (OR) of 0.94 (95% CI 0.89-0.98; *P*=.007) in favor of the PT + PN group. A new model, excluding the interaction term, showed a main effect of time (OR 0.93, 95% CI 0.91-0.95; *P*<.001) and no main effect of group (OR 1.42, 95% CI 0.92-2.20; *P*=.12). The difference between the groups based on average nights of use per week at week 12 was 0.9 nights per week in favor of the PT + PN group with a significant difference in OR of 0.39 (95% CI 0.21-0.72; *P*=.003).

#### CMS Adherence Threshold

**Figure 3:**
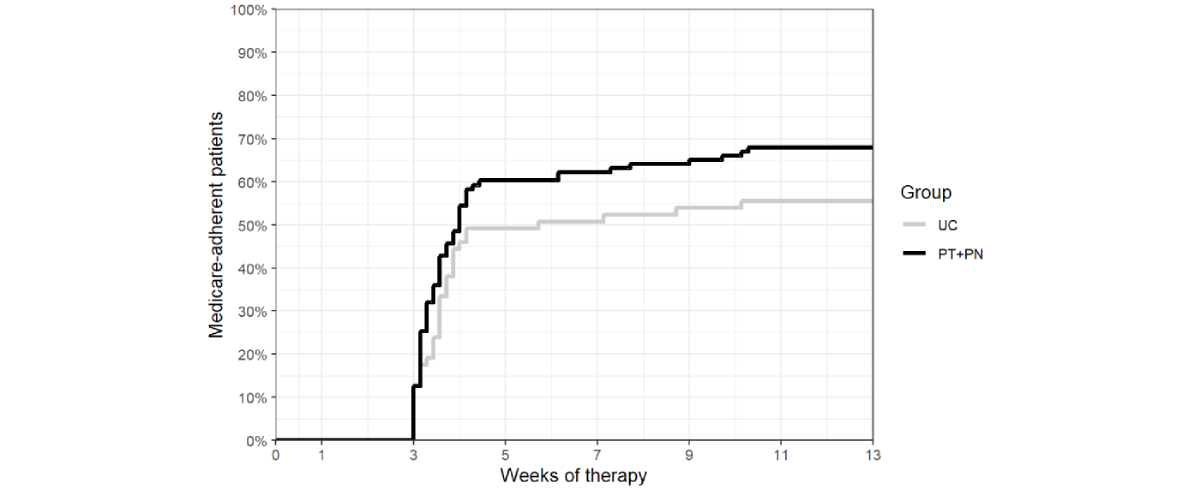
Kaplan-Meier curves showing the percentage of participants reaching the Centers for Medicare and Medicaid Services adherence threshold over time in the usual care (UC) group and personalized content in a tailored style + personalized content in a nontailored style (PT + PN) group.

The percentage of participants reaching the CMS adherence threshold was 68% (70/103) and 56% (35/63) in the PT + PN and UC groups, respectively, and the median number of days to reach the CMS adherence threshold was 28 and 41 in the PT + PN and UC groups, respectively. The Kaplan-Meier curves for the percentage of participants reaching the CMS adherence threshold over time for the PT + PN and UC groups (Figure 3) were not significantly different (log-rank test *χ*^2^_1_=2.4, *P*=.12).

#### Secondary Subjective Outcomes

The ESS scores, outcome expectations, perceived importance, motivation, self-efficacy, and therapy satisfaction results are shown in Table 2 for the PT + PN and UC groups and in Table 3 for the PT and PN groups. A comparison of the PT + PN and UC groups showed significant group-by-time interactions for outcome expectations (*P*=.02) and perceived importance (*P*<.001) in favor of the PT + PN group. No significant group-by-time interactions were observed for ESS, self-efficacy, motivation, or therapy satisfaction (all *P*>.05). There were main effects of time for ESS (*P*<.001), outcome expectations (*P*=.02), and motivation (*P*=.002), all with negative slopes, and a main effect of group for perceived importance in favor of the PT + PN group (*P*=.03).

**Table 2:** Secondary outcome measures across the study groups and the significant group-by-time interaction effects for the personalized content in a tailored style + personalized content in a nontailored style (PT + PN) versus usual care (UC) groups.

Outcome measure and group			Baseline		Day 11		Day 56		Day 90		Main effect of time					Main effect of group					Group-by-time interaction effect			
										95% CI			*P* value		95% CI			*P* value		95% CI			*P* value	
**ESS^a,b^ (on a scale of 24), mean (SD)**												−2.26 to −1.09		<.001^c^			−2.31 to 1.56		.70			−0.80 to 0.67		.86
	PT + PN	10.83 (5.44)		—^d^		7.63 (4.30)		7.40 (4.51)																
	UC	11.14 (5.59)		—		8.23 (4.42)		7.78 (4.43)																
**Outcome expectations (out of 5), mean (SD)**												−0.09 to −0.01		.02^c^			−0.02 to 0.40		.07			0.01 to 0.18		.02^c^
	PT + PN	4.41 (0.72)		4.05 (0.93)		4.31 (0.79)		4.32 (0.76)																
	UC	4.33 (0.72)		4.07 (0.65)		3.97 (0.88)		3.99 (1.03)																
**Perceived importance (out of 5), mean (SD)**												−0.05 to 0.02		.51			0.02 to 0.38		.03^c^			0.09 to 0.25		<.001^c^
	PT + PN	4.63 (0.66)		4.58 (0.79)		4.66 (0.68)		4.79 (0.52)																
	UC	4.65 (0.65)		4.62 (0.67)		4.36 (0.82)		4.31 (0.99)																
**Motivation (out of 10), mean (SD)**												−0.29 to −0.06		.002^c^			−0.40 to 062		.67			−0.08 to 0.21		.39
	PT + PN	9.45 (0.92)		8.95 (1.57)		9.31 (1.34)		9.02 (1.57)																
	UC	9.21 (1.40)		8.85 (1.47)		8.97 (1.57)		8.57 (1.83)																
**Self-efficacy (out of 5), mean (SD)**												−0.14 to 0.01		.08			−0.32 to 0.30		.96			−0.03 to 0.16		.20
	PT + PN	4.34 (0.72)		4.20 (0.83)		4.36 (0.79)		4.29 (0.93)																
	UC	4.29 (0.73)		4.07 (0.85)		4.14 (1.00)		4.04 (1.08)																
**Therapy satisfaction, mean (SD)**												−0.35 to 0.13		.35			−0.79 to 0.67		.86			−0.16 to 0.43		.37
	PT + PN	—		8.85 (1.58)		8.89 (1.58)		8.92 (1.63)																
	UC	—		9.00 (1.40)		8.57 (1.59)		8.63 (1.89)																

^a^ESS: Epworth Sleepiness Scale.

^b^Epworth Sleepiness Scale scores >10 correspond to excessive sleepiness.

^c^Statistically significant, *P*<.05

^d^Not assessed at this time point.

**Table 3:** Secondary outcome measures across the study groups and the significant group-by-time interaction effects for the personalized content in a tailored style (PT) versus personalized content in a nontailored style (PN) groups.

Outcome measure and group		Baseline	Day 11	Day 56	Day 90	Main effect of time				Main effect of group				Group-by-time interaction effects			
						95% CI		*P* value		95% CI		*P* value		95% CI		*P* value	
**ESS^a,b^ (on a scale of 24), mean (SD)**							−2.37 to −1.08		<.001^c^		−1.46 to 2.18		.70		−0.92 to 0.85		.95
	PT	10.42 (5.33)	—^d^	7.84 (3.97)	7.16 (3.95)												
	PN	11.18 (5.56)	—	7.45 (4.59)	7.60 (4.96)												
**Outcome expectations (out of 5), mean (SD)**							−0.10 to 0.06		.83		−0.36 to 0.23		.65		−0.10 to 0.10		.94
	PT	4.42 (0.63)	4.10 (0.85)	4.43 (0.70)	4.33 (0.73)												
	PN	4.39 (0.80)	4.01 (1.01)	4.26 (0.85)	4.30 (0.79)												
**Perceived importance (out of 5), mean (SD)**							−0.01 to 0.12		.10		−0.26 to 0.22		.88		−0.10 to 0.08		.84
	PT	4.69 (0.59)	4.53 (0.89)	4.65 (0.65)	4.86 (0.41)												
	PN	4.58 (0.71)	4.63 (0.72)	4.66 (0.71)	4.73 (0.60)												
**Motivation (out of 10), mean (SD)**							−0.26 to −0.03		.02^c^		−0.78 to 0.23		.28		−0.10 to 0.22		.47
	PT	9.59 (0.56)	9.10 (1.35)	9.38 (0.97)	9.11 (1.23)												
	PN	9.32 (1.14)	8.82 (1.75)	9.25 (1.59)	8.93 (1.81)												
**Self-efficacy (out of 5), mean (SD)**							−0.06 to 0.05		.89		−0.29 to 0.18		.65		0.02 to 0.24		.02^c^
	PT	4.49 (0.53)	4.26 (0.79)	4.29 (0.76)	4.28 (0.98)												
	PN	4.20 (0.84)	4.14 (0.86)	4.42 (0.81)	4.30 (0.89)												
**Therapy satisfaction, mean (SD)**							−0.15 to 0.19		.80		−0.53 to 0.48		.91		0.05 to 0.72		.02^c^
	PT	—	9.13 (1.29)	8.77 (1.72)	8.82 (1.81)												
	PN	—	8.61 (1.77)	8.98 (1.47)	9.00 (1.47)												

^a^ESS: Epworth Sleepiness Scale.

^b^Epworth Sleepiness Scale scores >10 correspond to excessive sleepiness.

^c^Statistically significant, *P*<.05.

^d^Not assessed at this time point.

### Effect of Tailoring the Style of the Personalized Intervention

#### Hours of Use Per Night

**Figure 4:**
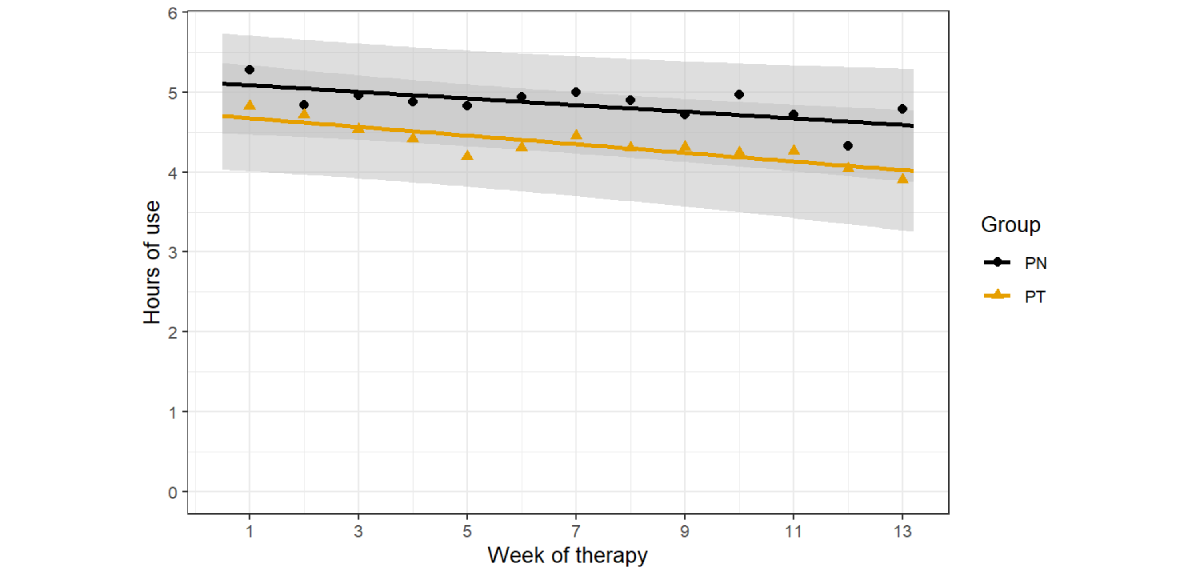
Average continuous positive airway pressure adherence (hours of use per night) in the personalized content in a tailored style (PT) and personalized content in a nontailored style (PN) groups. Each data point represents the mean adherence for each 7-day period. Each line is a fitted linear model with the shaded area corresponding to the 95% CI.

Average adherence throughout the 90-day study period was 4.35 (SD 3.13) hours per night in the PT group and 4.86 (SD 3.15) hours per night in the PN group. Figure 4 shows the average CPAP adherence in terms of hours of use per night in the PT and PN groups and the fitted linear model. The mixed model comparing the PT and PN groups showed that there was no significant group-by-time interaction, with an estimated difference in the slope of 0.11 minutes per night (95% CI −0.46 to 0.67; *P*=.71). The main effect of time was not significant (−0.36, 95% CI −0.74 to 0.03; *P*=.07), and there was no main effect of group (*P*=.38). The estimated difference in average use between the groups was 34.1 minutes per night on day 90 (95% CI −28.20 to 96.50; *P*=.28).

#### Nights of Use Per Week

**Figure 5:**
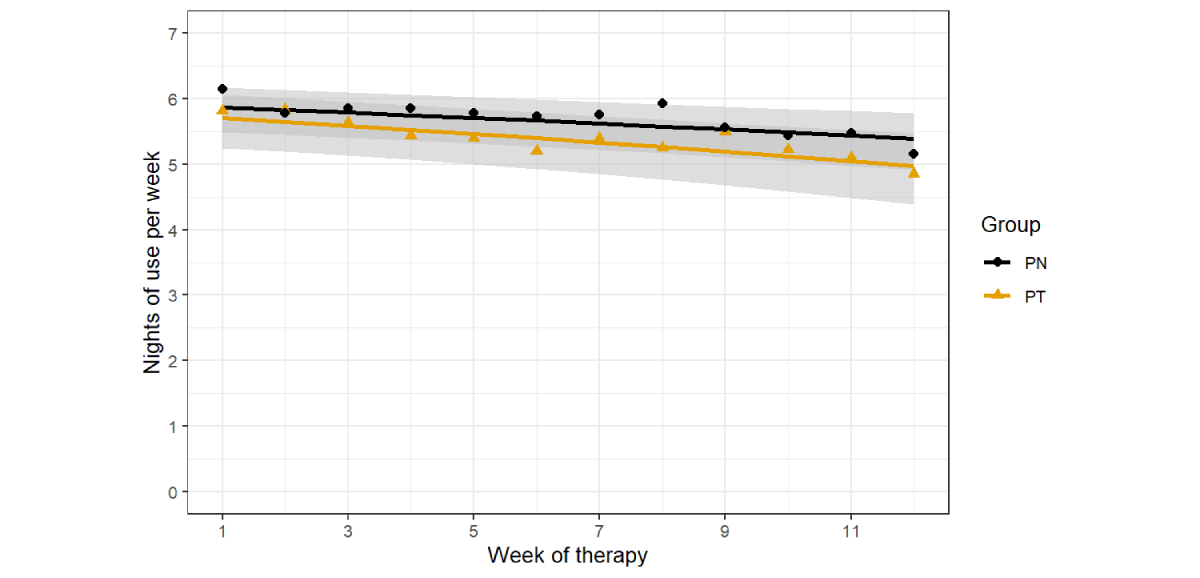
Average continuous positive airway pressure adherence (nights of use per week) in the personalized content in a tailored style (PT) and personalized content in a nontailored style (PN) groups. Each data point represents the mean adherence for each 7-day period. Each line is a fitted linear model with the shaded area corresponding to the 95% CI.

Figure 5 shows the average number of nights of CPAP use per week for the PT and PN groups. The PT and PN groups had an average weekly decline of 0.06 and 0.04 nights per week, respectively. The mixed-effects beta regression model showed no significant group-by-time interaction (difference in OR 0.99, 95% CI 0.95-1.02; *P*=.49). There was a main effect of time (difference in OR 0.96, 95% CI 0.94-0.99; *P*=.002) and no main effect of group (difference in OR 0.85, 95% CI 0.51-1.43; *P*=.054).

#### CMS Adherence Threshold

**Figure 6:**
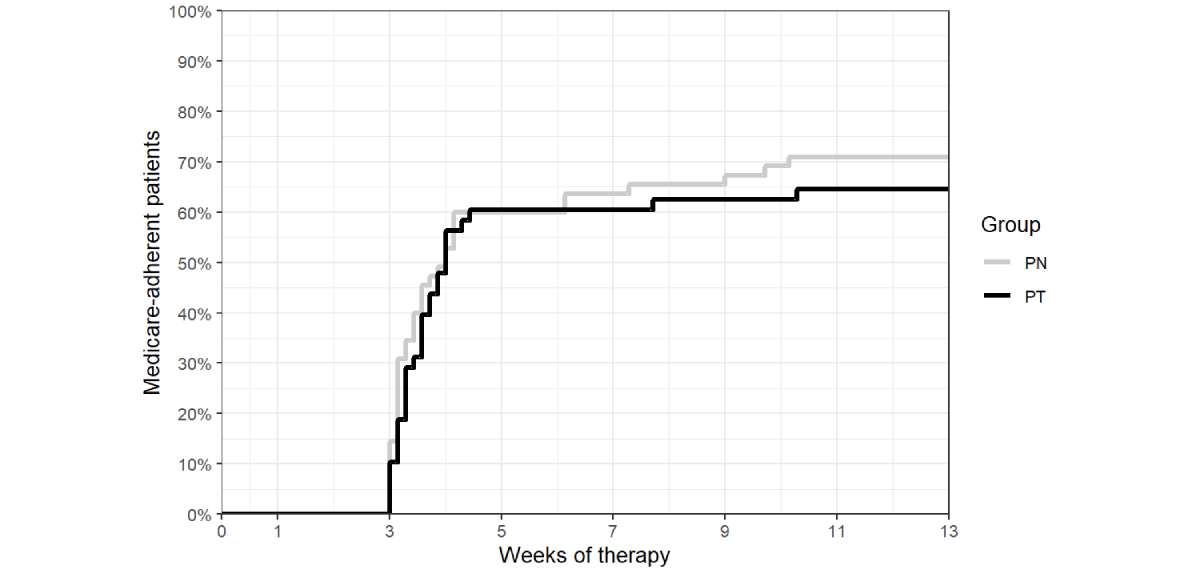
Kaplan-Meier curves showing the percentage of participants reaching the Centers for Medicare and Medicaid Services adherence threshold over time in the personalized content in a nontailored style (PN) and personalized content in a tailored style (PT) groups.

The Kaplan-Meier curves for the PT and PN groups (Figure 6) were not significantly different (log-rank test, *χ*^2^_1_=0.5, *P*=.50). The percentage of participants reaching the CMS adherence threshold during the study period was 65% (31/48) and 71% (39/55) in the PT and PN groups, respectively.

#### Secondary Subjective Outcomes

A comparison of the PT and PN groups showed a significant group-by-time interaction effect for self-efficacy (*P*=.02) and therapy satisfaction (*P*=.02) in favor of the PN group (Table 3). There were no other group-by-time interaction effects. There was a main effect of time for ESS (*P*<.001) and motivation (*P*=.02), both of which decreased over time; however, no other main effects of group or time were found.

## Discussion

### Principal Findings

We hypothesized that providing personalized CPAP education and feedback would be associated with increased adherence compared with UC. The results support this hypothesis. In addition, the intervention increased the outcome expectations and perceived importance of CPAP therapy, 2 psychological variables associated with CPAP use.

Furthermore, we hypothesized that adherence would increase when the content of the intervention was delivered in a tailored versus nontailored style. However, the results do not support this hypothesis. In addition, style tailoring did not improve the psychological variables associated with CPAP use.

In the subsequent paragraphs, we discuss the main findings.

The results showed a significant main effect of personalized feedback and education on CPAP adherence for the 2 primary outcomes: hours of use per night and nights of use per week.

There was a significant difference of 56.4 minutes (0.94 hours) of average use throughout the 90-day study period in favor of the group that received personalized feedback and education (PT + PN group) compared with the group that received UC (UC group). The rate of decline in nightly use was significantly smaller for the group that received personalized feedback and education (PT + PN group) than for the group that received UC (UC group), leading to a significant difference in the estimated average adherence based on minutes of use per night between the groups at study day 90 of 81.3 minutes in favor of the PT + PN group.

A similar pattern was observed for nights of use per week. There was a significant difference of 0.9 average nights of use per week throughout the 90-day study period in favor of the group that received personalized education and feedback (PT + PN group) compared with the group that received UC (UC group). A significant group-by-time interaction effect was observed in favor of the PT + PN group compared with the UC group. The average difference of 0.9 nights per week between the groups at week 12 was significant.

The results for the secondary adherence outcome measure of percentage of participants reaching the CMS adherence threshold showed that more participants in the PT + PN group reached the CMS adherence threshold than in the UC group, and the participants in the PT + PN group reached the threshold earlier, but the difference did not reach statistical significance.

Looking at the secondary outcome variables related to sleepiness (ESS) and the psychological factors associated with CPAP use (outcome expectations, perceived importance, motivation, self-efficacy, and therapy satisfaction), significant group-by-time interactions were obtained for outcome expectations and perceived importance in favor of the PT + PN group. No other group-by-time interactions were observed. A main effect of group for perceived importance showed that the PT + PN group, on average, scored significantly higher on perceived importance. Main effects of time for ESS (*P*<.001), outcome expectations (*P*=.02), and motivation (*P*=.002) showed that for both groups, these factors decreased significantly over time.

Taken together, the results underscore the positive effect of providing personalized feedback and education on CPAP adherence and thereby support our first hypothesis. However, we did not find similarly strong support for our second hypothesis that tailoring the style of the content of the intervention would further increase adherence.

Average adherence in terms of hours of use per night throughout the 90-day study period was not significantly different between the style-tailored (PT) and non–style-tailored (PN) groups. In addition, there was no group-by-time interaction effect and no main effect of group for the average nights of CPAP use per week for the PT and PN groups.

In line with the primary outcomes, no significant effect of style tailoring was observed on the secondary adherence outcome of the percentage of participants reaching the CMS adherence threshold.

Although no significant effects of style tailoring on adherence were obtained, significant group-by-time interaction effects of style tailoring were observed in favor of the PN group for 2 of the psychological factors: self-efficacy and therapy satisfaction. However, the differences in self-efficacy and therapy satisfaction between the groups were very small, and no differences were observed between the groups by the end of the study (day 90). In addition, there were no other main effects of group or time.

Taken together, we were unable to find support for our second hypothesis that tailoring the style of the personalized education and feedback intervention to the psychological profile of the patient has an effect on CPAP adherence.

### Clinical Significance of the Findings

It is widely accepted that a dose-response relationship exists between the hours of CPAP use per night and a range of clinical outcomes [7]. Quantifying the exact relationship has been proven challenging because the relationship differs according to clinical outcome, and the different clinical outcomes depend on many other variables that have often not been controlled for in the existing studies (eg, the individual habitual sleep duration). Two of the most common categories of outcomes that have been studied are sleep-related daytime symptoms (eg, sleepiness) and cardiovascular symptoms (eg, blood pressure).

A study examining the relationship between the average hours of CPAP use per night and sleepiness as well as daily functioning showed that among the participants who had excessive sleepiness before the start of CPAP treatment, those whose excessive sleepiness resolved with CPAP treatment had, on average, approximately 1 hour more CPAP use per night than those whose excessive sleepiness remained (5.1 vs 4.0 hours per night for self-reported sleepiness and 5.1 vs 3.9 hours per night for objective sleepiness) [39]. In addition, as for daily functioning, among the participants with functional impairment before the start of CPAP treatment, those who reached normal levels of daily functioning with CPAP treatment had, on average, 1 hour more CPAP use per night than those whose functional impairment remained (5.1 vs 4.1 hours per night).

Similar results were found in another study for self-reported sleepiness but not for objective sleepiness and daily functioning [40]. Finally, another study examining memory function found that the participants whose sleep-related memory impairment normalized with CPAP treatment had, on average, 1.6 hours more nightly CPAP use than those whose impairment did not normalize (5.2 vs 3.4 hours of use per night) [41].

CPAP adherence has also been associated with improvements in cardiovascular symptoms. The findings of several studies have shown a positive relationship between the average hours of CPAP use per night and blood pressure reduction in hypertensive patients with OSA starting CPAP treatment [42,43]. A meta-analysis including the data of 12 trials concluded that each additional hour of CPAP use per night was associated with a reduction of 1.39 mm Hg in mean blood pressure [44]. Another study examining the predictors of blood pressure improvements in patients with hypertension who had coronary artery disease and OSA identified CPAP adherence as one of the main independent predictors of decreases in blood pressure [45].

There is increasing evidence of a positive dose-response relationship between CPAP adherence and clinical outcomes. Although the assessment of clinical impact goes beyond the scope of this study, the impact of the intervention on CPAP adherence (a difference of 56.4 minutes on average throughout the 90-day study period and 81.3 minutes at study day 90) is comparable with the CPAP adherence differences shown to have clinical significance.

### Comparison With Clinical and eHealth Interventions

A review study investigating which intervention strategies are most effective in optimizing CPAP adherence in clinical settings concluded that the literature is inconclusive in this regard [7]. Clinics often offer a combination of education and support in the form of touch points with patients (eg, appointments or calls). Many clinical intervention studies used a combination of interventions and deployed strategies with various levels of intensity, making it difficult to interpret the individual effects. A study comparing standard and intensive interventions (including additional visits, telephone calls, and education) found that the patients who received intensive CPAP support had an average increase of 1.7 hours of nightly CPAP use compared with those who received standard, less intensive interventions [46].

In addition to education and support, some clinics have used other strategies, including the feeding back of personal sleep test data along with an explanation (eg, the patient viewing their own polysomnogram). Results from 3 studies using this strategy reported different average increases in nightly CPAP use: −0.2, 0.7, and 1.2 hours per night [22,47,48].

Despite the inconclusiveness in terms of the exact effective strategies, there is increasing consensus about the best practice recommendations that educational interventions be given with the initiation of therapy, that behavioral and troubleshooting interventions be given during the initial period of therapy, and that telemonitoring-guided interventions be used during the initial period of therapy [49]. Our intervention approach aligns with these recommendations given the provision of 2 educational brochures at the start and behavioral and troubleshooting interventions (weekly feedback reports with recommendations focused on stimulating and facilitating CPAP use, with special attention to constructs from theories of health behavior change) in the initial 90 days based on the personal telemonitored data of the patient.

In addition to clinical interventions that are often delivered by nurses or clinicians, health interventions are being increasingly delivered through digital means (ie, eHealth interventions). A recent meta-analytic review including 18 studies and 5429 adults with OSA investigating the effectiveness of a broad range of eHealth interventions aimed at improving CPAP treatment adherence concluded that eHealth interventions can improve adherence to CPAP in the initial months, increasing the average duration of use by 0.54 hours per night compared with 1.36 hours (81.3 minutes) per night in our study [50]. No significant differences in effects were observed between the studies that used eHealth as a stand-alone intervention and those that used eHealth in combination with human-delivered interventions. The review results and the results of our study underscore the potential of cost-effective alternatives to the labor-intensive interventions delivered by clinical staff.

### Strengths and Limitations of the Study

To the best of our knowledge, this is the first study to combine the personalization of education and feedback based on personal therapy data with tailoring the style of the intervention to the psychological characteristics of the patient to promote CPAP adherence in a population with OSA.

One of the core strengths of our study was that we worked closely with sleep clinics that invited patients, explained the study, onboarded the patients for the study, and guided the first assessments and delivery of the educational brochures. A motivated care professional who radiated the importance of the study may have had a positive impact on the adherence levels of the patients in the study.

Another potential strength was that the weekly CPAP reports were delivered to the participants in the PT and PN groups through a physical mail service. The provision of physical reports may have attracted more attention from the participants and thereby led to a more in-depth processing of the materials. When considering the cost-effectiveness of the intervention and the increasing number of people who use digital means for communication, the delivery of materials in a digital manner is a likely alternative to physical mail. Because the intervention materials are already generated digitally and are fully automated, the delivery of the materials using digital channels would turn the intervention into a fully digital intervention with lower delivery costs. It remains to be seen whether the effects obtained in this study will persist when physical reports are replaced with digital reports. A recent meta-analytic review investigating the effectiveness of a broad range of eHealth interventions in improving CPAP treatment adherence showed that digital interventions hold promise as an alternative to more labor-intensive or otherwise costly interventions [50]. Although the increase reported based on the meta-analytic review of eHealth interventions was smaller than the increase observed in our study, we cannot conclude that this was because the difference in delivery, that is, digital delivery versus mail delivery. We believe that it is worthwhile to explore a fully digital implementation of our intervention as a cost-effective alternative. As concluded by the authors, more evidence is needed to understand the timing, duration, intensity, and types of digital interventions that most effectively improve adherence. In line with the recommended best practices [49], our results suggest that education focused on behavioral constructs and stimulating and facilitating CPAP treatment and timely personalized feedback and recommendations based on personal telemonitored data may be effective components of a digital intervention. Policy makers and clinical organizations should consider these types of interventions as an alternative or add-on to the labor-intensive interventions delivered by clinical staff.

A limitation of our study is that we did not include stratification by mask type in our randomization procedure. Studies seem to have shown that oronasal masks have been associated with lower therapy effectiveness and lower adherence levels than other types of masks [51]. The UC group had a higher proportion of oronasal masks, which may have led to lower adherence levels in the UC group. However, although the PN group had a higher proportion of oronasal masks than the PT group, the average adherence levels for the PN group were not lower than those for the PT group (they were slightly but not significantly higher). Therefore, we expect that it is highly unlikely that mask type played a key role in explaining the differences in adherence levels between the groups.

Another limitation of our study is that the design did not allow us to examine the impact of style tailoring in isolation. The provision of style tailoring in combination with personalized education and feedback was examined and compared with the provision of personalized education and feedback without style tailoring. A possible explanation for the failure to find an additional positive effect of style tailoring on adherence levels is that the personalized education and feedback intervention already increased adherence levels substantially, thereby limiting the room for further impact. Alternatively, there may have been short-lasting small effects that failed to impact our outcome measures. In the domain of health psychology, there is evidence suggesting that the impact of a health-related message can be enhanced when the style of the message is tailored to psychological characteristics that relate to how the receiver perceives and processes information [52-56]. Nevertheless, the effects are subtle and often do not persist over extended periods. This finding may have been similar to that of our study.

Another explanation could be that the psychological profiling framework (ie, the model of the 4 profiles, the assessment and allocation to 1 of the 4 profiles, and the corresponding templates for tailoring the style of the intervention materials) that we used was simply not effectively addressing the information processing needs of the existing patient population. Although the profiles were obtained from extensive qualitative research with patients and nurses and the tailoring strategies were validated with patients and nurses, we have no data to substantiate that these profiles sufficiently matched the information needs of the patients in this specific study.

More and larger-scale research is needed to better understand the potential effects and specific implementation requirements that lead to a positive effect of style tailoring on adherence and adherence-related variables.

### Conclusions

The results of this randomized controlled trial showed that the provision of personalized education and feedback can increase adherence to CPAP therapy substantially. We were unable to show that tailoring the style of the intervention to the psychological profile of the patient further increased adherence.

Additional research is needed to investigate how the impact of interventions can be enhanced by catering to the differences in the psychological profiles of patients. In addition, future implementations should consider cost-effective ways of delivering the intervention through digital channels.

## Appendix

Multimedia Appendix 1 Profiling questionnaire.

Multimedia Appendix 2 Examples of the personalized continuous positive airway pressure (CPAP) reports delivered weekly.

Multimedia Appendix 3 CONSORT checklist.

## Abbreviations

CMS: Centers for Medicare and Medicaid
CPAP: continuous positive airway pressure
ESS: Epworth Sleepiness Scale
OR: odds ratio
OSA: obstructive sleep apnea
PN: personalized content in a nontailored style
PT: personalized content in a tailored style
UC: usual care
