# Supplementary material for: Effectiveness of an Intervention Providing Digitally Generated Personalized Feedback and Education on Adherence to Continuous Positive Airway Pressure: Randomized Controlled Trial
Source: J Med Internet Res. 2023 May 22;25:e40193. doi: 10.2196/40193 (PMC10242460; doi:10.2196/40193)
Supplement: Multimedia Appendix 1 [file jmir_v25i1e40193_app1.doc]

## Supplement 1: profiling questionnaire

There are different ways in which people cope with their chronic condition and their treatment. Below you can read 4 different statements.

Which statement applies to you THE MOST?

- I especially want to enjoy life, which makes me forget that the treatment is a part of my life.
- I need to know everything about my condition, so that I can make the right decisions about my treatment
- With my condition, I fully rely on my physician in offering me the best care and treatment
- I just want to know what to do and I will do it as long as it brings visible improvements

Below you can read the same statements again.

This time, we ask you which statement applies to you THE LEAST

- I especially want to enjoy life, which makes me forget that the treatment is a part of my life.
- I need to know everything about my condition, so that I can make the right decisions about my treatment
- With my condition, I fully rely on my physician in offering me the best care and treatment
- I just want to know what to do and I will do it as long as it brings visible improvements
