# Supplementary material for: Effectiveness of an Intervention Providing Digitally Generated Personalized Feedback and Education on Adherence to Continuous Positive Airway Pressure: Randomized Controlled Trial
Source: J Med Internet Res. 2023 May 22;25:e40193. doi: 10.2196/40193 (PMC10242460; doi:10.2196/40193)

## Supplement 2: Examples of personalized PAP reports delivered weekly

The examples of personalized PAP reports for the different profiles were created prior to study initiation for illustration purposes and do not contain any real personal data from any of the participants.

1. Fighter: front and back


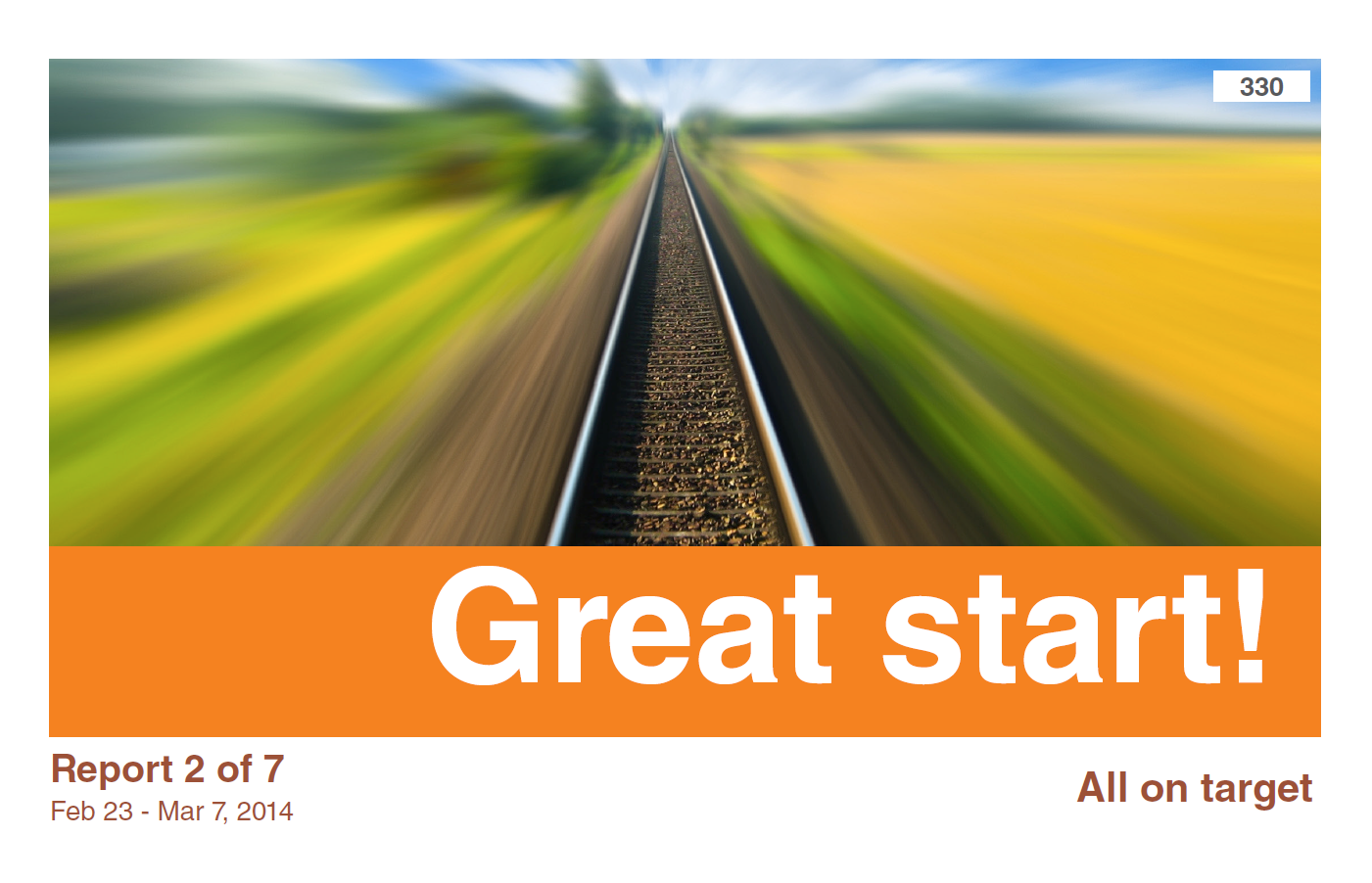

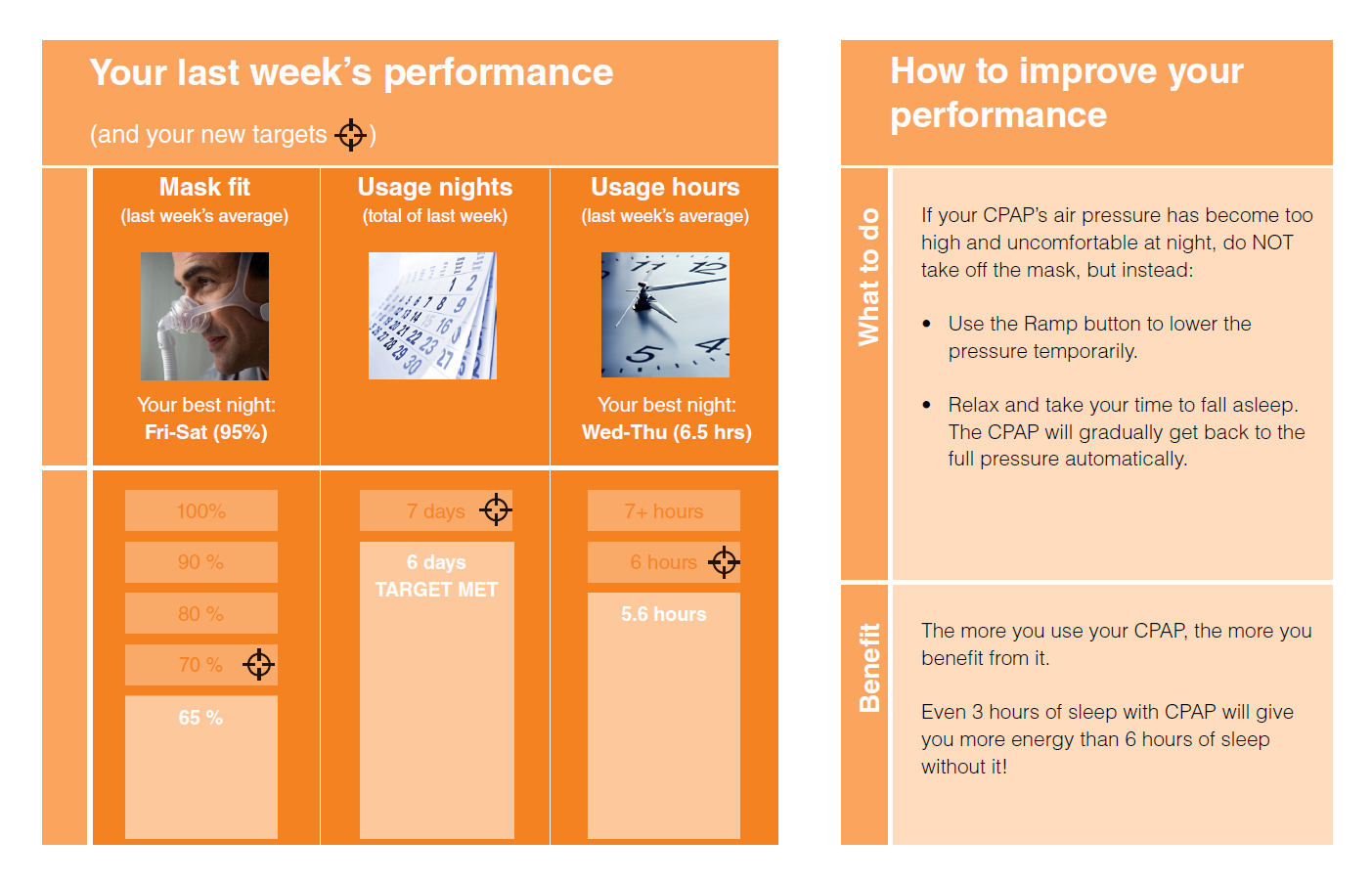


1. Analyst: front and back


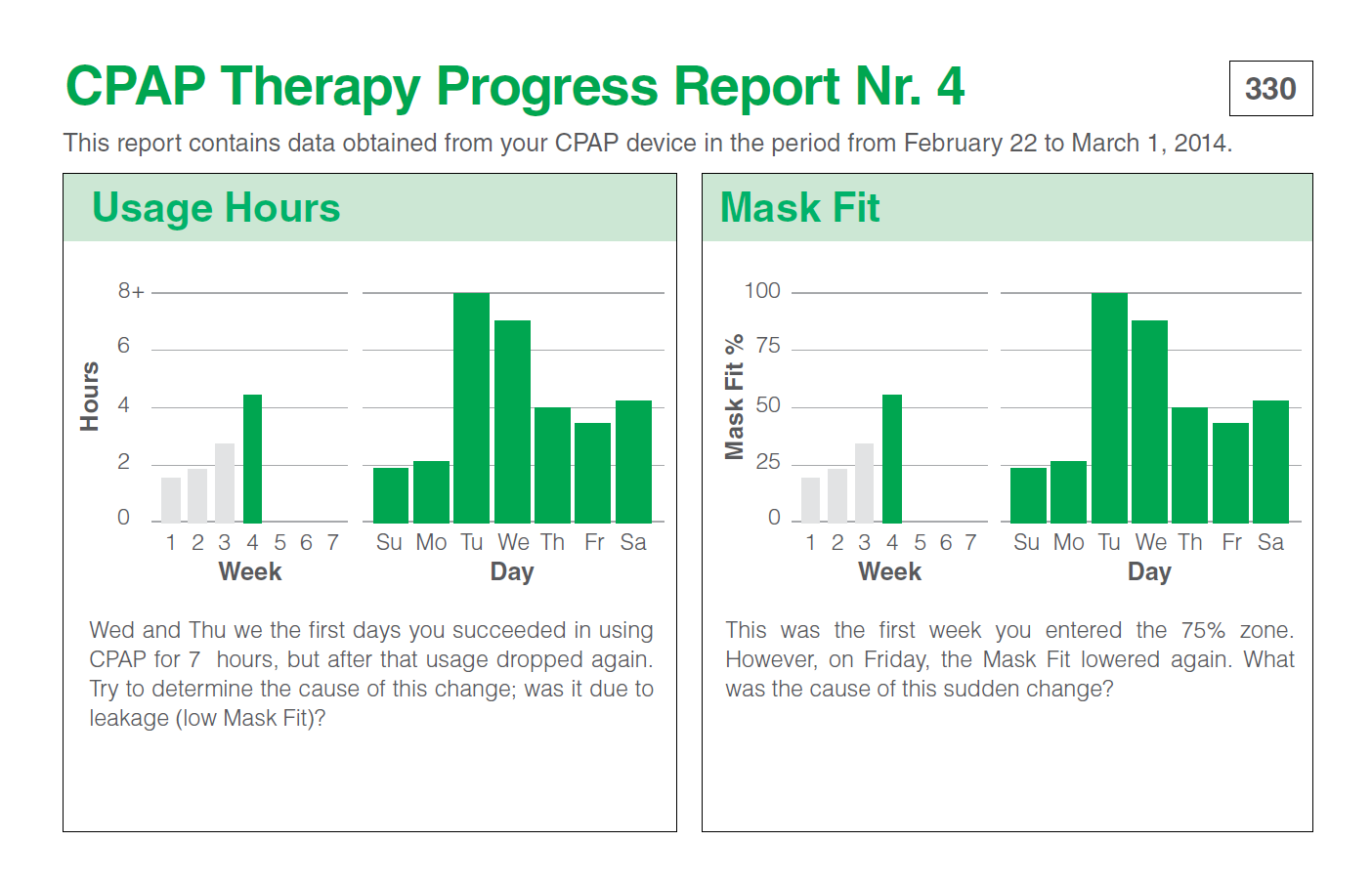

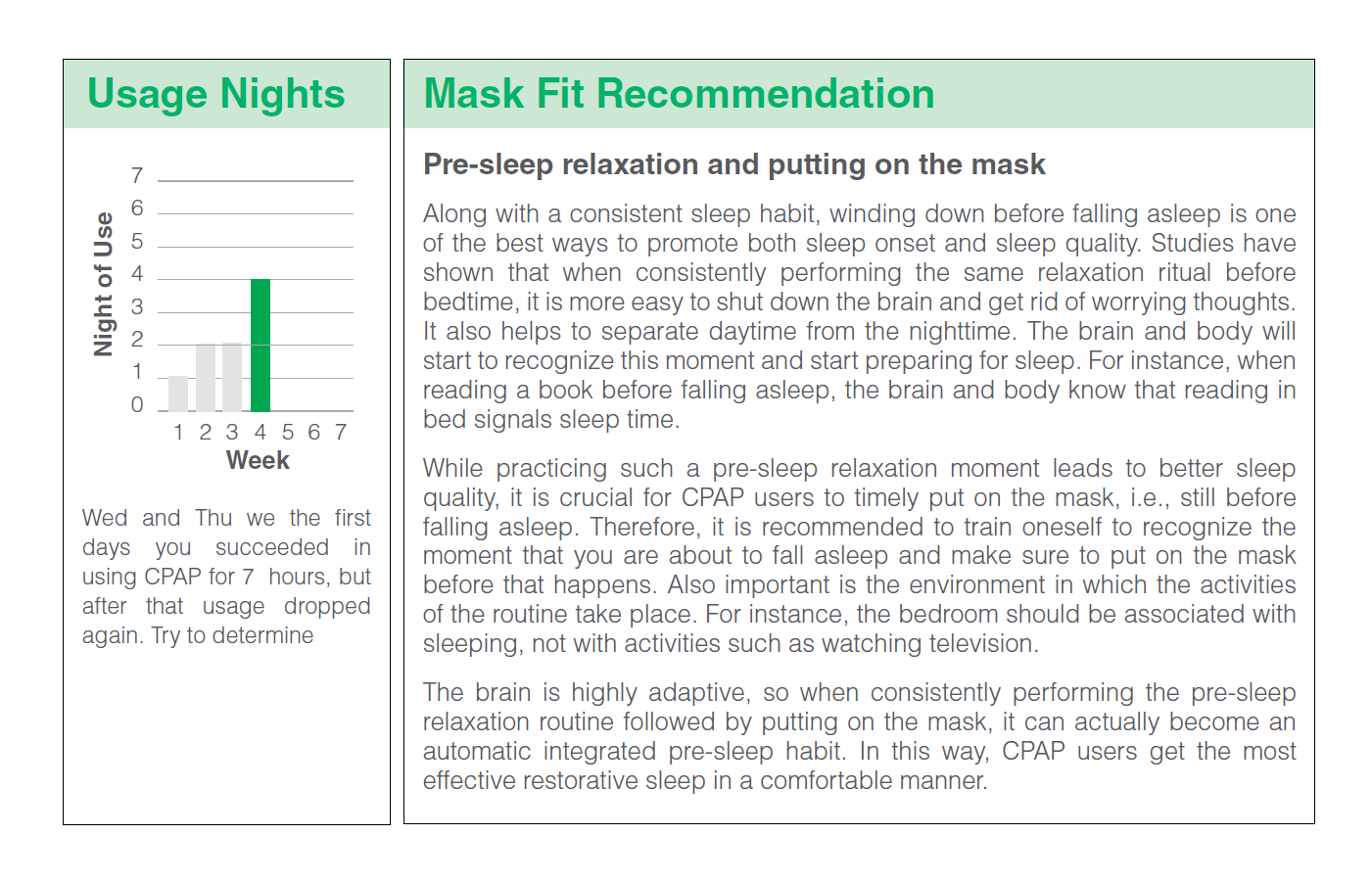


1. Sensitive: front and back


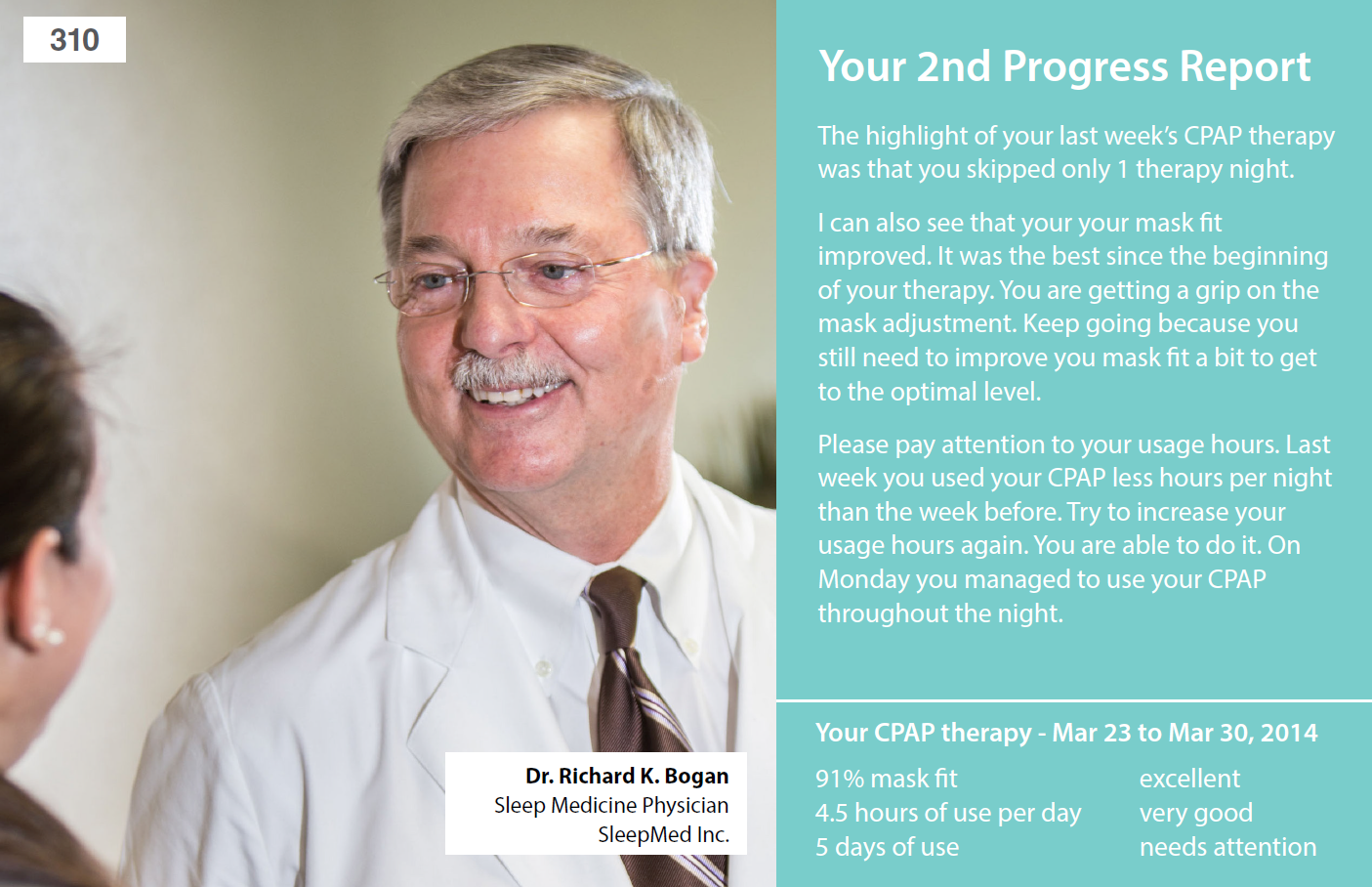

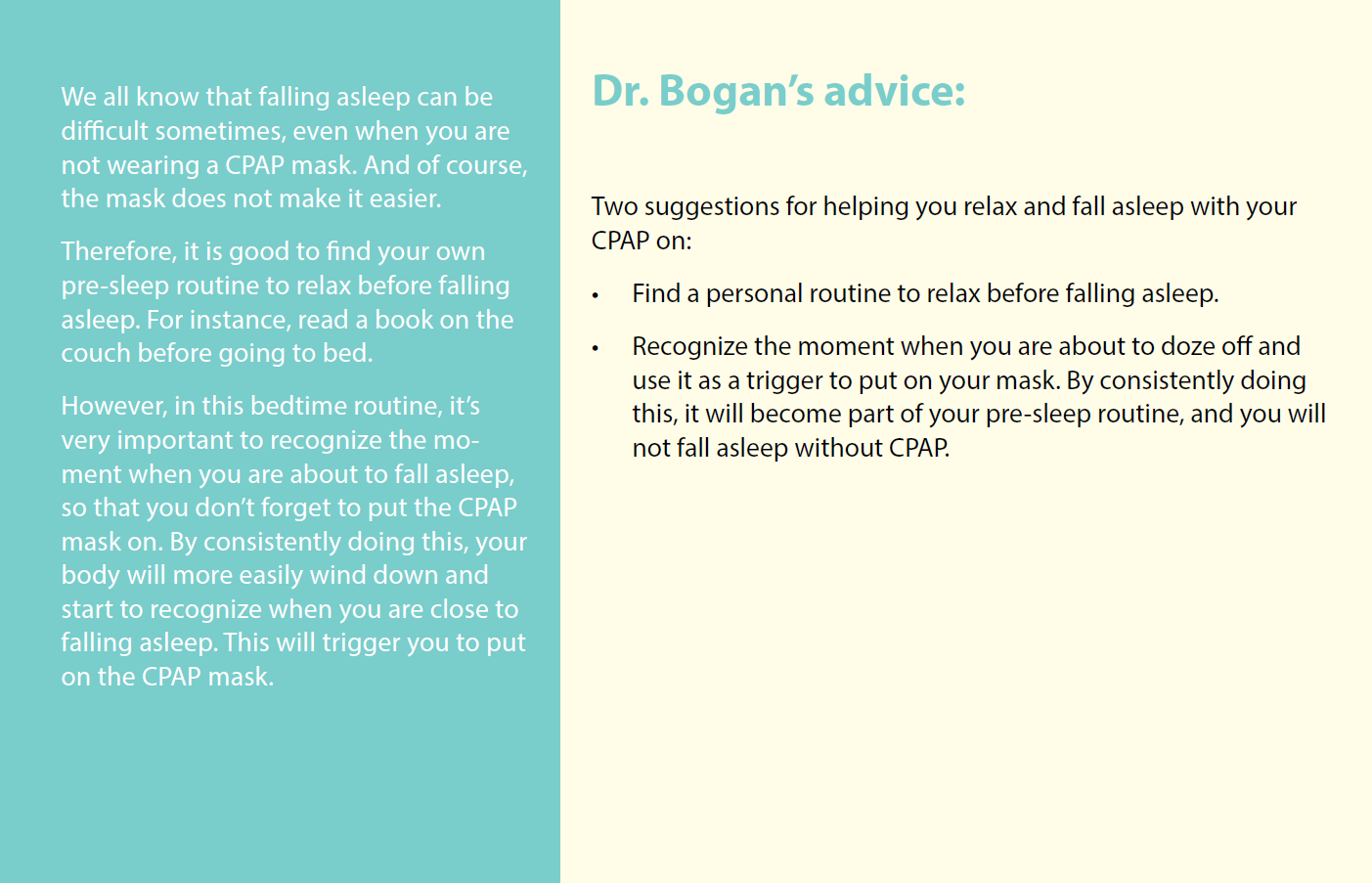


1. Optimist: front and back


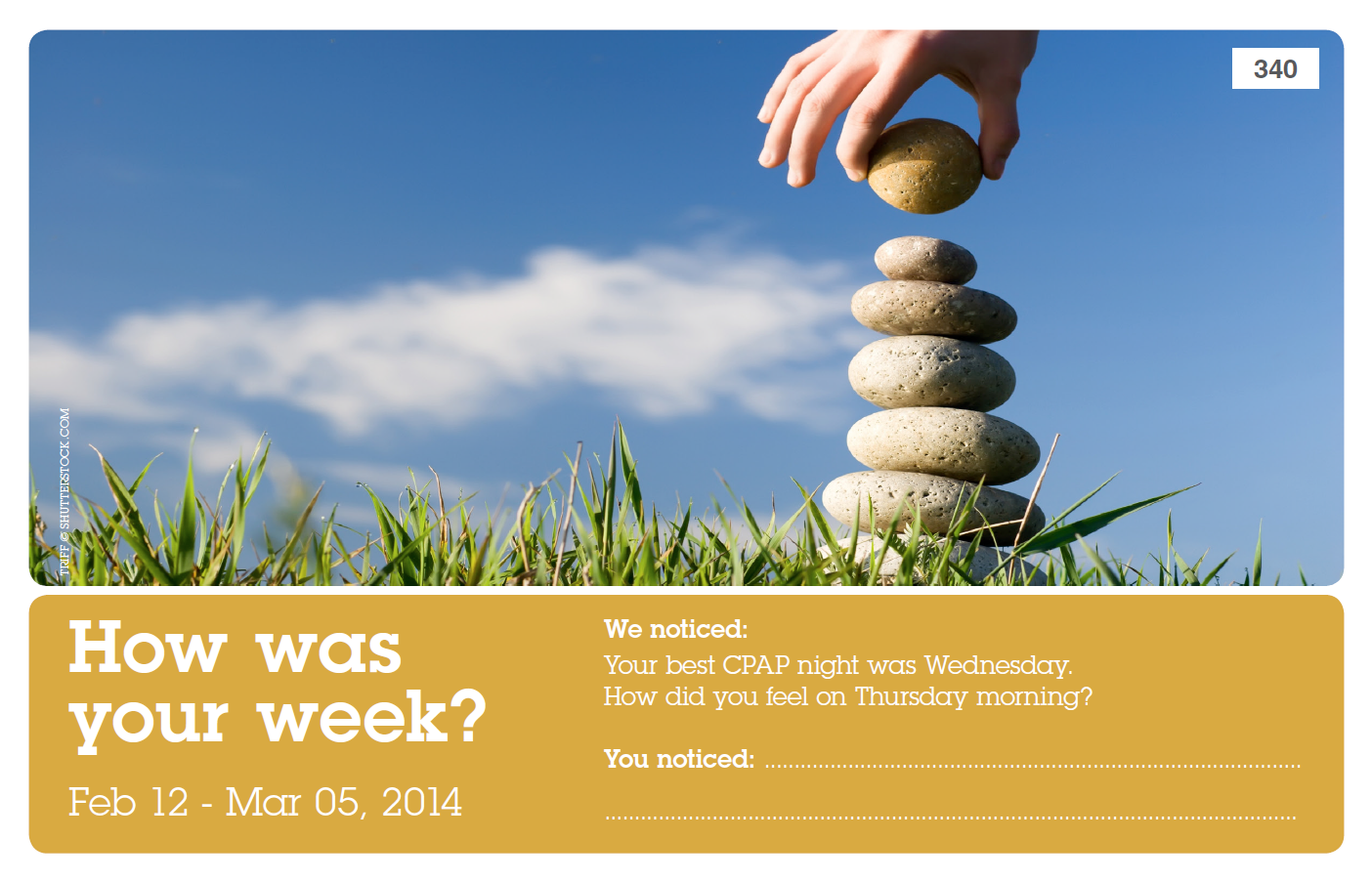

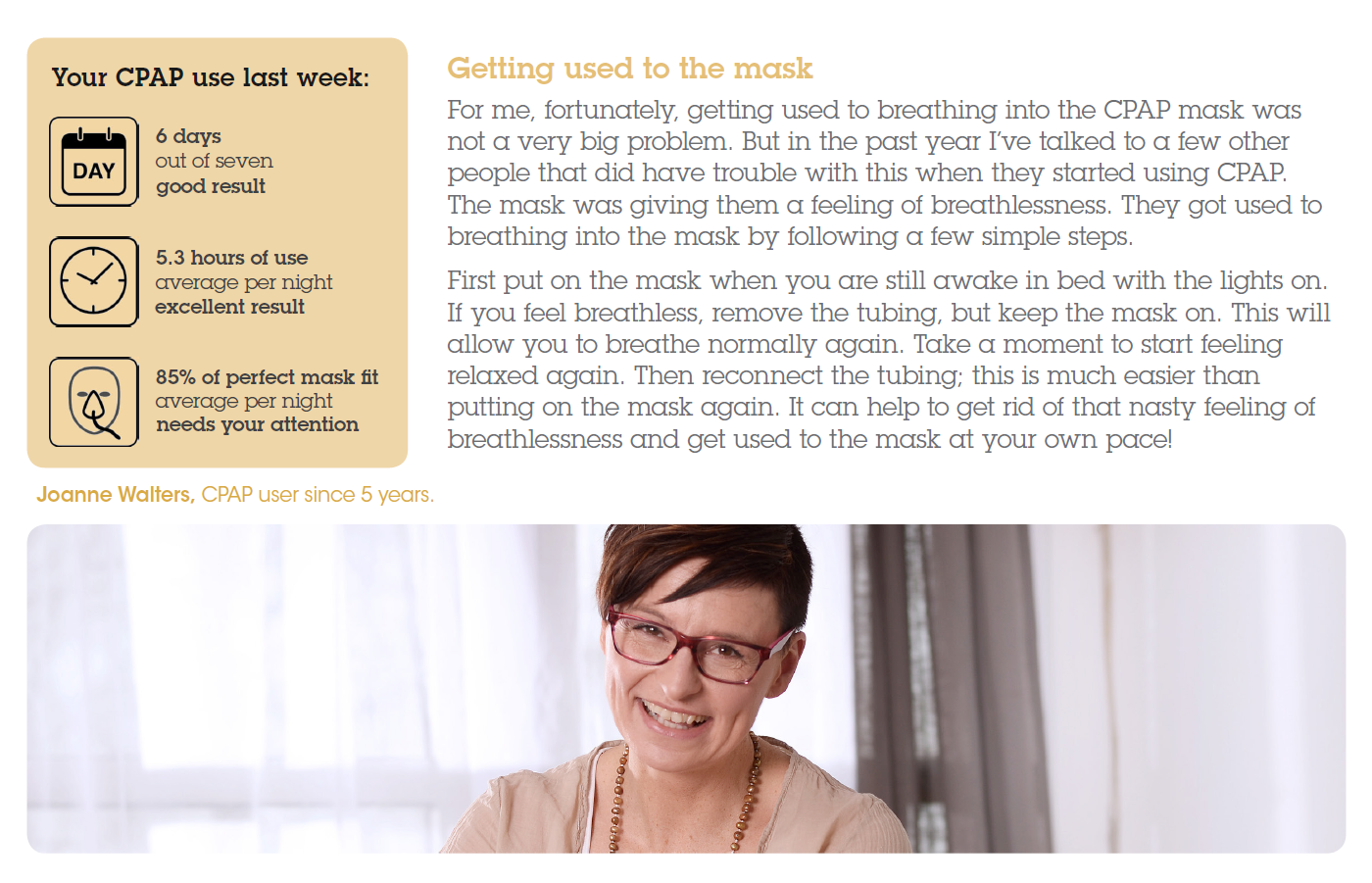

Supplement: Multimedia Appendix 2 [file jmir_v25i1e40193_app2.doc]
