# Supplementary material for: Effectiveness of an Intervention Providing Digitally Generated Personalized Feedback and Education on Adherence to Continuous Positive Airway Pressure: Randomized Controlled Trial
Source: J Med Internet Res. 2023 May 22;25:e40193. doi: 10.2196/40193 (PMC10242460; doi:10.2196/40193)
Supplement: Multimedia Appendix 3 [file jmir_v25i1e40193_app3.pdf]

Reporting checklist for randomised trial.

Based on the CONSORT guidelines.

		Reporting Item	Page Number
**Title and Abstract**			
Title	#1a	Identification as a randomized trial in the title.	In *Title*
Abstract	#1b	Structured summary of trial design, methods, results, and conclusions	In *Abstract*
**Introduction**			
Background and objectives	#2a	Scientific background and explanation of rationale	Section: *Introduction*
Background and objectives	#2b	Specific objectives or hypothesis	Section: *Introduction*
**Methods**			
Trial design	#3a	Description of trial design (such as parallel, factorial) including allocation ratio.	Section: *Design* and Section: *Recruitment, baseline assessment, randomization, and blinding*
Trial design	#3b	Important changes to methods after trial commencement (such as eligibility criteria), with reasons	n/a, no changes
Participants	#4a	Eligibility criteria for participants	Section: *Recruitment, baseline assessment, randomization, and blinding*
Participants	#4b	Settings and locations where the data were collected	Section: *Methods*

Interventions	#5	The experimental and control interventions for each group with sufficient details to allow replication, including how and when they were actually administered	Section: *Usual Care* and Section: *Adherence Intervention*
Outcomes	#6a	Completely defined prespecified primary and secondary outcome measures, including how and when they were assessed	Section; *Assessment of outcomes*
Outcomes	#6b	Any changes to trial outcomes after the trial commenced, with reasons	n/a, no changes
Sample size	#7a	How sample size was determined.	Section: *Power*
Sample size	#7b	When applicable, explanation of any interim analyses and stopping guidelines	n/a, no interim analyses or stopping guidelines
Randomization - Sequence generation	#8a	Method used to generate the random allocation sequence.<br><br>Section: *Recruitment, baseline assessment, randomization, and blinding*	
Randomization - Sequence generation	#8b	Type of randomization; details of any restriction (such as blocking and block size)<br><br>Section: *Recruitment, baseline assessment, randomization, and blinding*	
Randomization - Allocation concealment mechanism	#9	Mechanism used to implement the random allocation sequence (such as sequentially numbered containers), describing any steps taken to conceal the sequence until interventions were assigned	Section: *Recruitment, baseline assessment, randomization, and blinding*

Randomization - Implementation	#10	Who generated the allocation sequence, who enrolled participants, and who assigned participants to interventions	Section: *Recruitment, baseline assessment, randomization, and blinding*
Blinding	#11a	If done, who was blinded after assignment to interventions (for example, participants, care providers, those assessing outcomes) and how.	Section: *Recruitment, baseline assessment, randomization, and blinding*
Blinding	#11b	If relevant, description of the similarity of interventions	n/a, not relevant
Statistical methods	#12a	Statistical methods used to compare groups for primary and secondary outcomes	Section: *Statistical Analysis*
Statistical methods	#12b	Methods for additional analyses, such as subgroup analyses and adjusted analyses	n/a, no additional analyses were performed on top of the predefined analyses
**Results**			
Participant flow diagram (strongly recommended)	#13a	For each group, the numbers of participants who were randomly assigned, received intended treatment, and were analysed for the primary outcome	Section: *Results* (described, no flow diagram)
Participant flow	#13b	For each group, losses and exclusions after randomization, together with reason	Section: *Results*
Recruitment	#14a	Dates defining the periods of recruitment and follow-up	Section: *Methods*
Recruitment	#14b	Why the trial ended or was stopped	n/a, the trial was not stopped unplanned
Baseline data	#15	A table showing baseline demographic and clinical characteristics for each group	*Table 1*
Numbers analysed	#16	For each group, number of participants (denominator) included in each analysis	Section: *Results*

		and whether the analysis was by original assigned groups	
Outcomes and estimation	#17a	For each primary and secondary outcome, results for each group, and the estimated effect size and its precision (such as 95% confidence interval)	Section: *Results*
Outcomes and estimation	#17b	For binary outcomes, presentation of both absolute and relative effect sizes is recommended	n/a, no binary outcomes
Ancillary analyses	#18	Results of any other analyses performed, including subgroup analyses and adjusted analyses, distinguishing pre-specified from exploratory	n/a, all analyses performed were pre-specified
Harms	#19	All important harms or unintended effects in each group (For specific guidance see CONSORT for harms)	n/a, no adverse events or unintended effects
**Discussion**			
Limitations	#20	Trial limitations, addressing sources of potential bias, imprecision, and, if relevant, multiplicity of analyses	Section: *Strengths and limitations of the study*
Generalisability	#21	Generalisability (external validity, applicability) of the trial findings	Section: *Comparison with clinical and eHealth interventions*
Interpretation	#22	Interpretation consistent with results, balancing benefits and harms, and considering other relevant evidence	Section: *Clinical significance of the results*
Registration	#23	Registration number and name of trial registry	Section: *Methods*
**Other information**			
Interpretation	#22	Interpretation consistent with results, balancing benefits and harms, and considering other relevant evidence	Section: *Clinical significance of the results*

Registration	#23	Registration number and name of trial registry	Section: *Methods*
Protocol	#24	Where the full trial protocol can be accessed, if available	n/a
Funding	#25	Sources of funding and other support (such as supply of drugs), role of funders	Section: *Conflicts of Interest*

None The CONSORT checklist is distributed under the terms of the Creative Commons Attribution License CC-BY. This checklist can be completed online using https://www.goodreports.org/, a tool made by the EQUATOR Network in collaboration with Penelope.ai
